# MFG-E8 accelerates wound healing in diabetes by regulating “NLRP3 inflammasome-neutrophil extracellular traps” axis

**DOI:** 10.1038/s41420-020-00318-7

**Published:** 2020-09-10

**Authors:** Wei Huang, Jinyu Jiao, Ju Liu, Meng Huang, Yanyan Hu, Wenzhuo Ran, Li Yan, Yin Xiong, Mei Li, Ziyao Quan, Yahua Rao, Jiayi Chen, Yan Huang, Dongxin Zhang

**Affiliations:** 1 Department of Laboratory Medicine, Wuhan No.1 Hospital, Tongji Medical College, Huazhong University of Science and Technology, Wuhan, Hubei China; 2 Department of Cardiac Function, Wuhan Asian Heart Hospital, Wuhan University of Science and Technology, Wuhan, Hubei China; 3 Department of Geriatrics, Wuhan No. 1 Hospital, Tongji Medical College, Huazhong University of Science and Technology, Wuhan, Hubei China; 4 Department of Dermatology, Wuhan No.1 Hospital, Tongji Medical College, Huazhong University of Science and Technology, Wuhan, Hubei China; 5 Clinical Laboratory of Medicine, Hubei University of Chinese Medicine, Wuhan, China; 6 Department of Clinical Laboratory, Wuhan Fourth Hospital, Puai Hospital, Tongji Medical College, Huazhong University of Science and Technology, Wuhan, Hubei China

**Keywords:** Diabetes complications, Inflammasome, Cell death and immune response

## Abstract

Sustained activation of NLRP3 inflammasome and release of neutrophil extracellular traps (NETs) impair wound healing of diabetic foot ulcers (DFUs). Our previous study reported that milk fat globule epidermal growth factor VIII (MFG-E8) attenuates tissue damage in systemic lupus erythematosus. However, the functional effect of MFG-E8 on “NLRP3 inflammasome-NETs” inflammatory loop in wound healing of diabetes is not completely elucidated. In this study, neutrophils from DFU patients are susceptible to undergo NETosis, releasing more NETs. The circulating levels of NET components neutrophil elastase and proteinase 3 and inflammatory cytokines IL-1β and IL-18 were significantly elevated in DFU patients compared with healthy controls or diabetic patients, in spite of higher levels of MFG-E8 in DFU patients. In *Mfge8*^*−/−*^ diabetic mice, skin wound displayed exaggerated inflammatory response, including leukocyte infiltration, excessive activation of NLRP3 inflammasome (release of higher IL-1β, IL-18, and TNF-α), largely lodged NETs, resulting in poor angiogenesis and wound closure. When stimulated with high-dose glucose or IL-18, MFG-E8-deficient neutrophils release more NETs than WT neutrophils. After administration of recombinant MFG-E8, IL-18-primed NETosis of WT or *Mfge8*^*−/−*^ neutrophils was significantly inhibited. Furthermore, NET and mCRAMP (component of NETs, the murine equivalent of cathelicidin LL-37 in human)-mediated activation of NLRP3 inflammasome and production of IL-1β/IL-18 were significantly elevated in *Mfge8*^*−/−*^ macrophages compared with WT macrophages, which were also significantly dampened by the administration of rmMFG-E8. Therefore, our study demonstrated that as inhibitor of the “NLRP3 inflammasome-NETs” inflammatory loop, exogenous rMFG-E8 improves angiogenesis and accelerates wound healing, highlighting possible therapeutic potential for DFUs.

## Introduction

Wound healing is impaired in diabetes, and diabetic foot ulcers (DFUs) with high morbidity, high disability, and high mortality, are seriously endangering the health of human^1^. Neutrophils are the main leukocytes involved in the early phase of healing. For microbial defense, neutrophils died by NETosis and released their nuclear and granular contents known as neutrophil extracellular traps (NETs)^2^. However, NETs can also induce tissue damage, particularly in diabetes, in which neutrophils are more susceptible to NETosis^3^. Using proteomics, NET components (elastase, histones, neutrophil gelatinase-associated lipocalin, and proteinase 3) were elevated in the blood of patients with DFUs. High concentration of NETs in the wound was associated with infection and a subsequent worsening of DFUs. Moreover, neutrophils isolated from the blood of DFU patients showed an increased spontaneous NETosis^4^. Diabetes has inflammatory or metabolic components such as high glucose that predispose neutrophils to NETosis^5^. Under diabetic conditions, reactive oxygen species, cytokines also induced NET formation^6^. TNF-α stimulates neutrophils to NETosis, which is elevated in diabetic patients^7,8^. NETosis begins with the activation of peptidylarginine deiminase 4 (PAD4), an enzyme leads to histone citrullination and chromatin decondensation^9^. Wong et al. elucidated that skin PAD4 activity was increased by diabetes, and inhibiting NETosis by Cl-amidine (PAD4 inhibitor) and cleaving NETs with DNase I may improve wound healing and reduce NET-driven chronic inflammation in diabetes^3,4^. Thus, NETosis is detrimental for wound healing in subjects with diabetes.

During impaired healing associated with diabetes, macrophages exhibits sustained NACHT, LRR, and PYD domains-containing protein 3 (NLRP3) inflammasome activity and release of IL-1β and IL-18^10^. Further studies showed that serum IL-18 concentrations were elevated in DFU patients, and inhibition of inflammasome activation improves the impaired pattern of healing in diabetes^11,12^. NET formation was driven in part by IL-1β, which demonstrated that activation of NLRP3 inflammasome was associated with proinflammatory NETs release^13^. It also has been founded that IL-18 primed human neutrophils for NETosis, in turn, enhanced formation of NETs could lead to increase NLRP3 inflammasome activation in macrophages, resulting in a feed-forward inflammatory loop^14^. In diabetes, the activation of NLRP3 inflammasome machinery in macrophages promotes inflammation and NETosis, in turn, NETs contribute to a self-perpetuating cycle of enhanced IL-1β and IL-18 production, leading to accumulation of NETs and inflammatory mediators in wound site and impair wound healing.

Milk fat globule epidermal growth factor VIII (MFG-E8) is a glycoprotein that interacted with phagocytes to stimulate the uptake of the apoptotic cells, and is ubiquitously expressed in various organs and cells^15,16^. In addition to its critical role in efferocytosis, MGF-E8 induced resolution of wound inflammation, improvements in angiogenesis, and acceleration of wound closure. During cutaneous ischemia–reperfusion injury, MFG-E8 may inhibit the formation of pressure ulcer by regulating angiogenesis and inflammation^17^. However, in diabetic condition, hyperglycemia and exposure to advanced glycated end products inactivated MFG-E8, recognizing a key mechanism that complicates diabetic wound healing^18^.

Further studies founded that MFG-E8 administration was associated with decreased NLRP3 inflammasome activation, and inhibited IL-1β production through mediation of integrin β3 and P2X_7_ receptor interactions in post-ischemic cerebral injury^19,20^. In addition, our previous study revealed that neutrophil recruitment and NETosis were enhanced in MFG-E8 knockout mice, showing that MFG-E8 attenuated the formation of NET in systemic lupus erythematosus^21^. However, the detailed mechanisms for MFG-E8 regulating wound healing of DFU patients remain elusive. In this work, we sought to characterize whether MFG-E8 regulates “NLRP3 inflammasome-NETs” loop to promote wound healing in diabetes.

## Results

### NET formation and NLRP3 inflammasome targets IL-1β/IL-18 in patients with DFUs

**Fig. 1:**
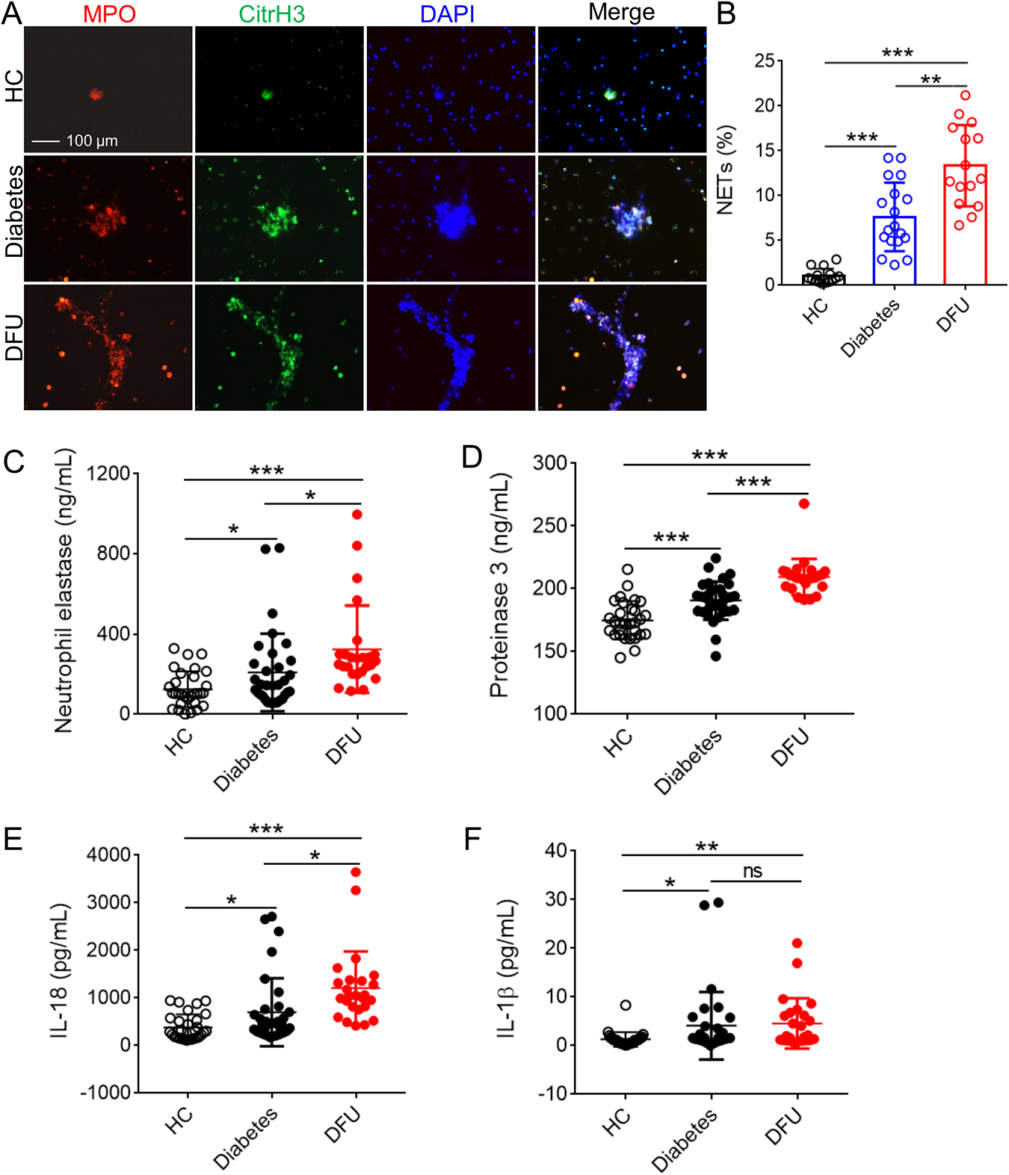
The activation of NLRP3 inflammasome and NETosis in patients with DFUs. **a** Neutrophils were purified from peripheral blood of HCs (*n* = 10), diabetes (*n* = 12), and DFU patients (*n* = 12) under spontaneous NETosis without FBS cultivation; and visualization of NETs with citrullinated histone H3 (CitrH3), MPO, and DNA. Three colors were merged by software Image J (NIH), original magnification ×200. **b** NET% was calculated in neutrophils from HCs, diabetic, and DFU patients (NET% = positive enlarged nuclei/total neutrophils per field, and the average of NET release ratio was evaluated >500 cells in more than five fields per sample were counted. **c**–**f** Serum neutrophil elstase **c**, proteinase 3 **d**, IL-18 **e**, and IL-1β **f** concentration in HCs (*n* = 30), diabetes (*n* = 33), and DFU patients (*n* = 25) were detected with ELISA. For all experiments, data are presented as mean ± SEM, *P < 0.05, **P < 0.01, ***P < 0.001, ns = not significant.

Under diabetic conditions, neutrophils produce more superoxide and cytokines than normoglycemic conditions. To test whether neutrophils from DFU patients primed to NETosis, we isolated the neutrophils from fresh whole blood obtained from patients with diabetes and DFUs whose glycated hemoglobin was >6.5%, indicating mild prolonged hyperglycemia, and from healthy individuals as controls (Table S1). Immunofluorescence assay showed that neutrophils from patients with diabetes or DFUs were more susceptible to prime NETosis spontaneously than healthy controls, and neutrophils from DFU patients released higher NETs than from diabetic patients (Fig. 1a, b). As expected, the levels of NET components such as neutrophil elastase (NE) and proteinase 3 (PR3) were elevated in patients with DFUs than diabetic patients, and both of them were higher than healthy controls (Fig. 1c, d). Moreover, serum levels of IL-1β and IL-18 were significantly higher in both diabetic and DFU patients than in healthy individuals; of interest, DFU patients showed levels higher than diabetic patients (Fig. 1e, f). Thus, neutrophils from DFU patients prime NETosis spontaneously, and the increased NETs and activation of NLRP3 inflammasome is associated with impaired wound healing.

### **MFG-E8 deficiency impairs wound closure and vascularization**

**Fig. 2:**
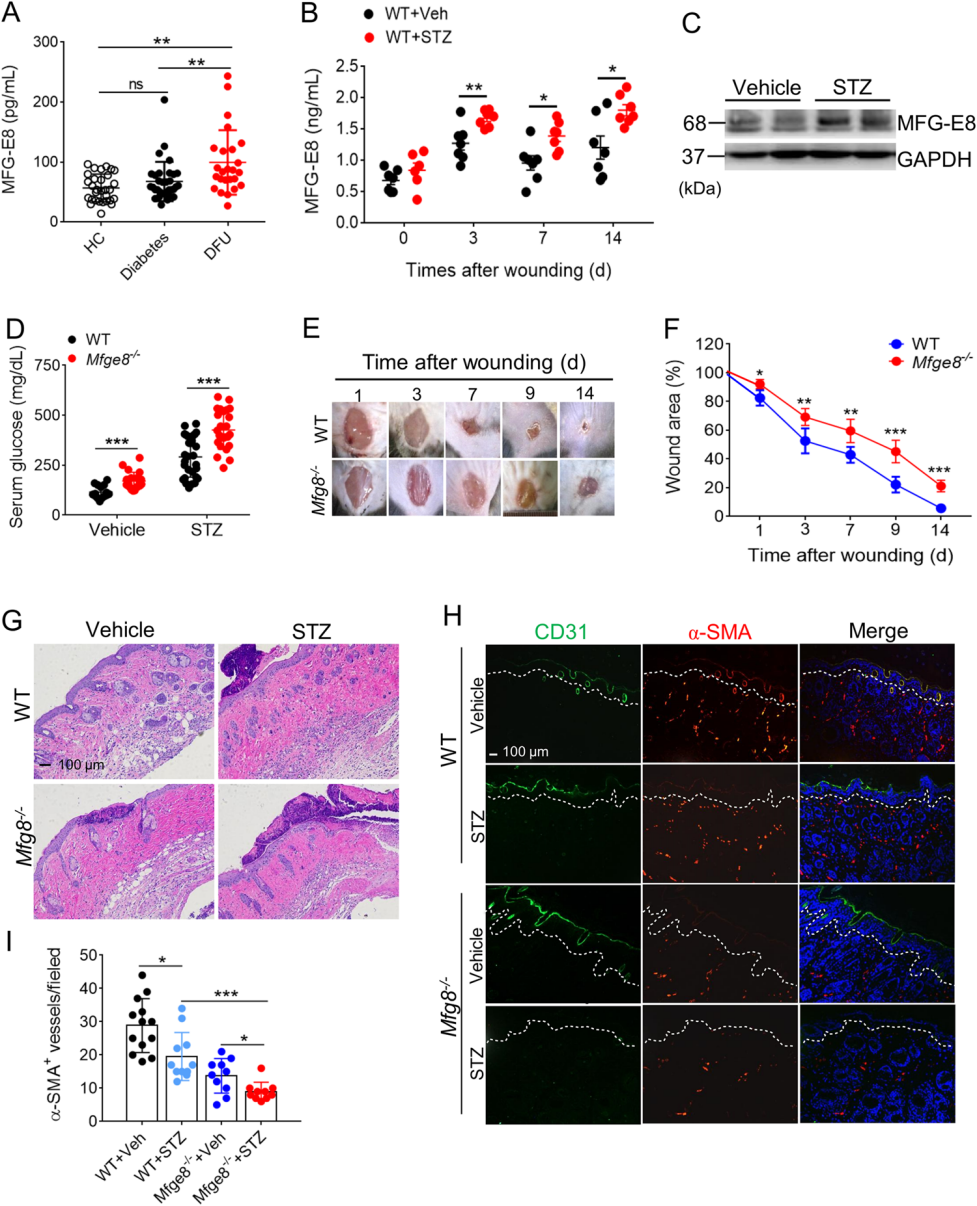
MFG-E8 deficiency impairs wound closure and wound angiogenesis. **a** The serum levels of MF-E8 in HCs (*n* = 30), diabetes (*n* = 33), and DFU patients (*n* = 25) were detected with ELISA. **b** The serum levels of MFG-E8 in normal or diabetic mice (*n* = 6) post-wounding for 0, 3, 7, 14 days were determined by ELISA. **c** Western blot analysis of MFG-E8 expression in skin tissues of normal or diabetic mice after wound for 3 days, GAPDH as a loading control. **d** The serum fed glucose levels of WT (*n* = 24) or *Mfge8*^*−/−*^ mice (*n* = 28) when treated with vehicle or STZ for 28 days were measured. **e** Representative digital imaging of wounds from age-matched diabetic WT or *Mfge8*^*−/−*^ mice postwounding up to day 14. **f** Wound closures kinetics of diabetic WT and *Mfge8*^*−/−*^ mice (*n* = 6). **g** Representative images of H&E-stained skin tissues from WT or *Mfge8*^*−/−*^ mice injected with vehicle or STZ after wound for 3 days. **h** The amount of CD31^+^ epithelial cells (ECs) and α-smooth muscle actin (α-SMA^+^) pericytes in wound skins of WT or *Mfge8*^*−/−*^ mice treated with vehicle or STZ at day 14 after wound. **i** Quantification of α-SMA^+^ vessels in five random microscopic fields in *n* = 6 mice per group was performed. For all experiments, data are presented as mean ± SEM, *P < 0.05, **P < 0.01, ***P < 0.001.

MFG-E8 serves as bridging molecule between apoptotic cells and phagocytes enabling efferocytosis^15^, which induced resolution of wound inflammation, improvements in angiogenesis, and acceleration of closure^18^. To investigate the effect of MFG-E8 on the wound healing in DFU patients, serum levels of MFG-E8 in healthy individuals, diabetic and DFU patients were analyzed. Data displayed that serum levels of MFG-E8 were increased in patients with DFUs as compared with healthy controls and diabetic patients (Fig. 2a). Accordingly, the serum levels of MFG-E8 were remarkably elevated in streptozocin (STZ)-induced diabetic mice than normal mice postwounding 3, 7, or 14 day (Fig. 2b). Wound-edge tissues were collected at day 3, 7, 14 postwounding to study the abundance of MFG-E8. A significant increase in wound-edge MFG-E8 protein was noted on day 3 postwounding in diabetic mice as compared with normal mice, as well as higher apoptotic cell accumulation (Figs. 2c and S1).

To further explore the underlying mechanism of MFG-E8 in wound healing, wounds were created on the dorsal surface of the hind limb in WT and *Mfge8*^*−/−*^ mice with STZ-induced diabetes. The mean levels of fed glucose in peripheral blood of *Mfge8*^*−/−*^ mice induced by STZ were significantly higher as compared with WT mice, and the proportion of successfully induced diabetic mice in *Mfge8*^*−/−*^ mice by STZ was higher than that in WT mice, accompanying with severe weight loss (Figs. 2d and S2). Insulin-positive islets were broken in pancreas of mice induced with STZ, particularly in *Mfge8*^*−/−*^ mice (Fig. S3), suggesting that extensive failure of insulin-positive islets was the factor of high levels of glucose in STZ-induced *Mfge8*^*−/−*^ mice. Moreover, diabetic *Mfge8*^*−/−*^ mice exhibited significantly delayed wound healing as compared with WT mice. By day 9, ~80% of the wounds on diabetic WT mice were fully closed, whereas ~50% of the wounds on diabetic *Mfge8*^*−/−*^ mice were healed (Fig. 2e, f). H&E staining confirmed that re-epithelialization was significantly impaired and infiltrated inflammatory cells, mainly neutrophils, were accumulated in wound skin tissues of *Mfge8*^*−/−*^ mice on day 3–14 postwounding as compared with that of WT mice, which overlaped with keratinocyte proliferation stage (Fig. 2g). To identify the vascularization of wound skin by immunofluorescence, we found that CD31^+^ epithelial cells (ECs) and αSMA^+^ (α-smooth muscle actin) pericytes/vascular smooth muscle cells were reduced in healing skin tissues of diabetic *Mfge8*^*−/−*^ mice (Fig. 2h, i). These results suggest that an important angiogenic role of MFG-E8 in wound healing.

### The absence of MFG-E8 enhances diabetic skin inflammation after wound

**Fig. 3:**
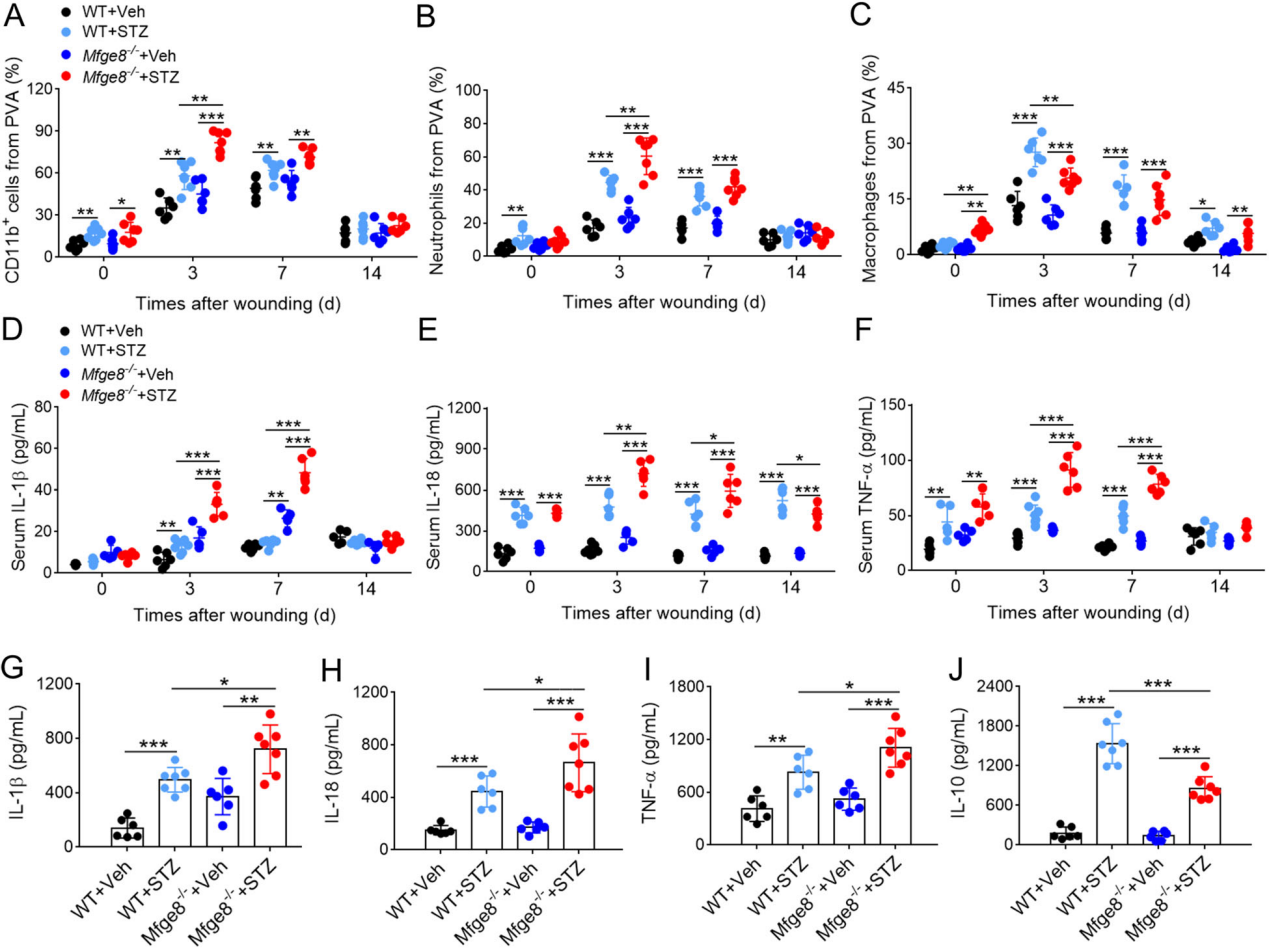
MFG-E8 deficiency enhances inflammatory response at the wound sites of diabetic mice. **a**–**c** Statistical analysis of total CD11b^+^ cells **a**, CD11b^+^ Ly6G^+^ neutrophils **b**, and CD11b^+^ F4/80^+^ macrophages **c** in PVA wound sites of WT or *Mfge8*^*−/−*^ (*n* = 6) mice treated with vehicle or STZ post-wounding at day 0, 3, 7, and 14. **d**–**f** Inflammatory cytokine profiles response to wound. The serum levels of IL-1β **d**, IL-18 **e**, and TNF-α **f** from WT or *Mfge8*^*−/−*^ mice (*n* = 6) treated with vehicle or STZ post-wounding at day 0, 3, 7, and 14 were measured with ELISA. **g**–**j** Cytokine profiles in wound site. The concentration of IL-1β **g**, IL-18 **h**, TNF-α **i**, and IL-10 **j** in PVA from wound skins of WT or *Mfge8*^*−/−*^ mice (*n* = 6) treated with vehicle or STZ post-wounding at day 3 were determined with ELISA. For all experiments, data are presented as mean ± SEM, *P < 0.05, **P < 0.01, ***P < 0.001.

The numbers of neutrophils and macrophages in wound fluid were quantified at day 0, 3, 7, and 14 postwounding of WT and *Mfge8*^*−/−*^ mice treated with vehicle or STZ. In diabetic mice, the leukocytes subsets recruited in wound skin tissues after wound were mainly composed by CD11b^+^ cells. The *Mfge8*^*−/−*^ mice showed an increase in total CD11b^+^ cells in the wound fluids at day 3 as compared with that in WT mice treated with STZ (Fig. 3a). At day 3 postwounding, the number of neutrophils in the wound fluids of diabetic *Mfge8*^*−/−*^ mice was significantly elevated than that in WT mice (Fig. 3b). Macrophages were major contributors to cutaneous wound healing, which were markedly decreased in diabetic *Mfge8*^*−/−*^ mice as compared with diabetic WT mice at day 3 postwounding (Fig. 3c). Accordingly, the serum levels of IL-1β, IL-18, and TNF-α were significantly increased in diabetic *Mfge8*^*−/−*^ mice postwounding 3 or 7 day than that in WT mice (Fig. 3d–f). The NLRP3 inflammasome activation has been implicated in the pathogenesis of diabetic wound lesion, and our results indicated significant increase of IL-18, IL-β, and TNF-α production in wound fluids of diabetic *Mfge8*^*−/−*^ mice at day 3 postwounding as compared with diabetic WT mice (Fig. 3g–i). Consistently, wound edge of *Mfge8*^*−/−*^ mice displayed a decrease secretion of anti-inflammatory IL-10 (Fig. 3j). These data indicated that the activation of inflammasome and production of IL-18/IL-1β may be delayed factors of wound healing in diabetic *Mfge8*^*−/−*^ mice.

### MFG-E8-deficient mice showed higher activation of NLRP3 inflammasome in wound site

**Fig. 4:**
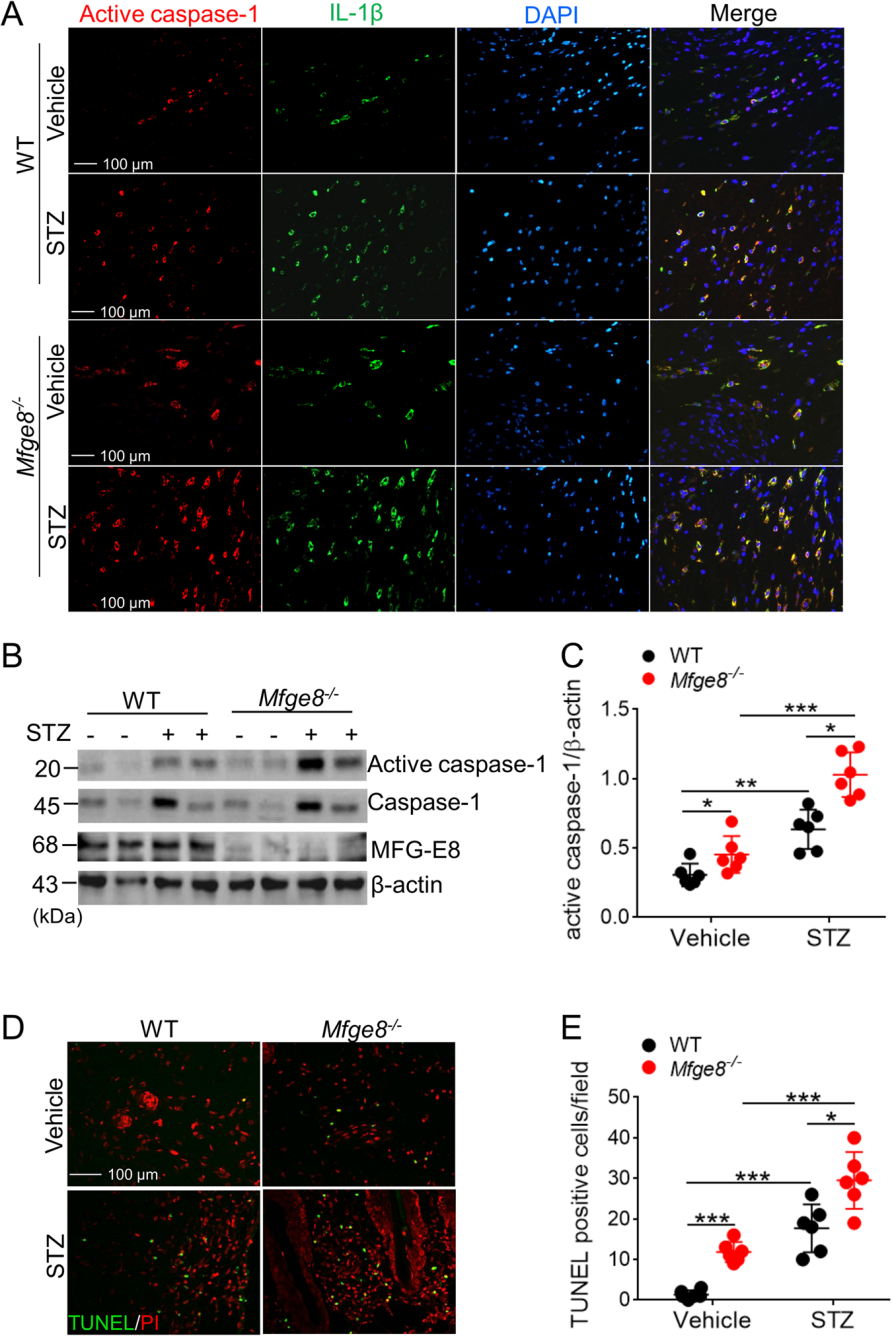
The activation of NLRP3 inflammasome was aggravated in MFG-E8-deficient mice. **a** The infiltrated active caspase-1^+^ and IL-1β^+^ macrophages in dermis of wound skins from WT or *Mfge8*^*−/−*^ mice (*n* = 6) treated with vehicle or STZ, post-wounding at day 3, were determined with immunofluorescence. **b** Quantification of the expression of active caspase-1, caspase-1, and MFG-E8 in wound skin tissue from WT or *Mfge8*^*−/−*^ mice (*n* = 6) treated with vehicle or STZ at day 3 post-wounding, β-actin as loading control. **c** Calculation of the ratio of active caspase-1/caspase-1 in wound skin tissues from each group mice. **d** Infiltration of death cells into wound skin tissues of WT or *Mfge8*^*−/−*^ mice (*n* = 6) post-wounding at day 3 was identified by TUNEL. Representative images were shown, original magnification ×200. **e** The average TUNEL-positive cells were counted at more than five random microscopic fields. For all experiments, data are presented as mean ± SEM, *P < 0.05, **P < 0.01, ***P < 0.001.

The activation of NLRP3 inflammasome and production of IL-18/IL-1β have important role in diabetic wound healing^10^. Immunofluorescence showed that the expression of activated caspase-1 and IL-1β were significantly elevated in wound skins of diabetic *Mfge8*^*−/−*^ mice as compared with WT mice (Fig. 4a). Western blot data also demonstrated that the expression of caspase-1 and active caspase-1 were enhanced in wound skins of diabetic *Mfge8*^*−/−*^ mice than that in WT mice (Fig. 4b, c). Following with activation of NLRP3 inflammasome and pyroptosis, higher density of dead cells accumulated in the wound skins of diabetic *Mfge8*^*−/−*^ mice (Fig. 4d, e), which were not being efficiently engulfed by MFG-E8-deficient macrophages^21^.

### **Diabetes prime MFG-E8-deficient neutrophils to undergo NETosis**

**Fig. 5:**
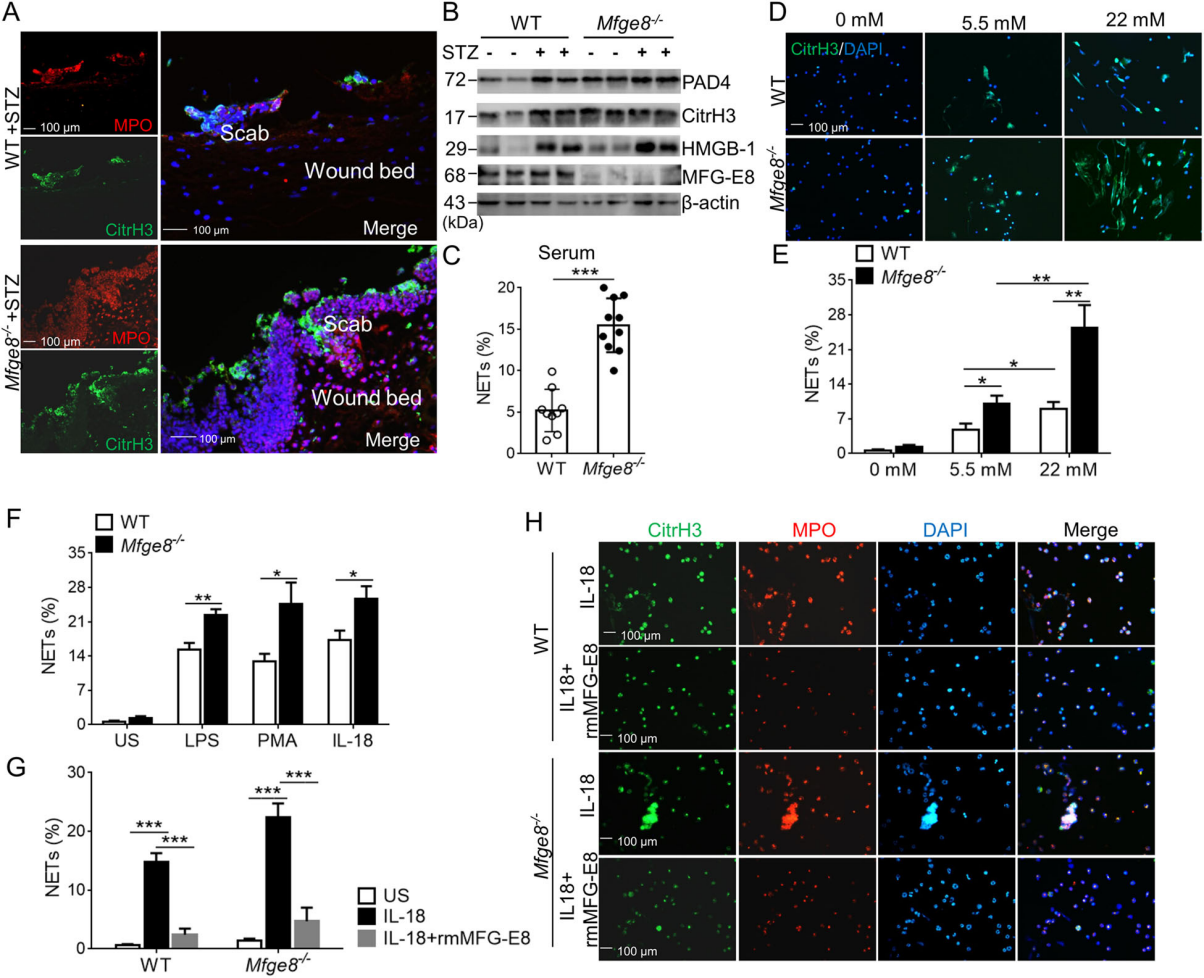
MFG-E8 attenuates NETosis in diabetic wound mice. **a** Immunofluorescence images of NETs in the wound bed beneath the scab from diabetic WT or *Mfge8*^*−/−*^ mice (*n* = 6) after wound for 3 days, visualized with DNA, CitrH3, and MPO. Three color were merged by software Image J (NIH). **b** Western blot of the wound tissues collected post-wounding at day 3 from WT or *Mfge8*^*−/−*^ mice (*n* = 6) treated with vehicle or STZ, to detect the expression of PAD4, CitrH3, HMGB-1, and MFG-E8, β-actin as loading control. **c** WT neutrophils primed NETosis, stimulated with the serum from diabetic WT or *Mfge8*^*−/−*^ mice (*n* = 6) post-wounding at day 3, and the percentage of NET formation were calculated. **d** Representative immunofluorescence images of NETs from isolated neutrophils of WT or *Mfge8*^*−/−*^ mice (*n* = 6) exposed to different glucose concentrations in vitro. **e** Percentage of produced NETs in neutrophils from WT or *Mfge8*^*−/−*^ mice (*n* = 5) after exposure with normal or high glucose were displayed. **f** The in vitro NET release of WT or *Mfge8*^*−/−*^ neutrophils (*n* = 5) was detected after stimulation with PMA, LPS, or IL-18. US, unstimulated. **g** After treatment with 500 ng/mL rmMFG-E8, the NET% were calculated in WT or *Mfge8*^*−/−*^ neutrophils (*n* = 5) stimulated with IL-18. **h** The representative images of NET release from isolated WT or *Mfge8*^*−/−*^ neutrophils (*n* = 5) induced by IL-18. For all experiments, data are presented as mean ± SEM, *P < 0.05, **P < 0.01, ***P < 0.001.

Our previous study showed that MFG-E8-deficient neutrophils released high levels of NETs, which lodged in damaged tissues of mice with SLE, demonstrating that NETs can be injurious to tissues^21^. Immunofluorescence analysis revealed an increase amount of citrullinated histone H3 (citrH3)- and myeloperoxidase (MPO)-merged NETs in wounds of diabetic Mfge8^−/−^ mice than that in WT mice, which were present in the wound bed immediately beneath the scab, of note, NETs were absent in the surface layers of unwounded skin (Fig. 5a). Wound skin expresses higher levels of PAD4, citrH3, high mobility group protein B1 (HMGB-1) in diabetic *Mfge8*^*−/−*^ mice after 3 days postwounding as compared with WT mice (Fig. 5b). A variety of stimuli could prime neutrophils to NETosis, including pathogens, proinflammatory cytokines IL-1β, TNF-α, PMA (phorbol 12-myristate 13-acetate), and nitric oxide^22^. After stimulation with serum from wound diabetic *Mfge8*^*−/−*^ mice, neutrophils formed higher NETs than that from WT mice (Fig. 5c), owing to the increase secretion of IL-1β, IL-18, and TNF-α (Fig. 3d–f). When compared to exposure with normal glucose, neutrophils released more NETs exposed to high glucose (Fig. 5d, e), particularly in *Mfge8*^*−/−*^ neutrophils, indicating a possible priming role of high glucose in *Mfge8*^*−/−*^ mice, who showed higher levels of serum glucose after treatment with STZ (Fig. 2a). Thus, the diabetic mice represent well the human condition with respect to susceptibility to NETosis, in particularly, diabetic *Mfge8*^*−/−*^ mice showed increased NETs lodged in injury tissues and impaired wound healing.

As previous report illustrated that IL-18 can stimulate NET formation, we examined whether MFG-E8 deficiency affect IL-18-induced NET release. After stimulation with recombinant mouse IL-18, MFG-E8-deficient neutrophils form high levels of NETs than WT neutrophils, which was equivalent to what has been reported for two well-characterized NETosis stimuli, PMA, and LPS (Fig. 5f). Furthermore, treatment with recombinant mouse MFG-E8, reduced NETosis of WT or *Mfge8*^*−/−*^ neutrophils stimulated with IL-18 (Fig. 5g, h). These observation suggest that activated NLRP3 inflammasome targeting IL-1β and IL-18 in wound sites of diabetes primed NET formation from recruited neutrophils, which were abrogated by exogenous MFG-E8.

### **NETs stimulate the activation of NLRP3 inflammasome and release of IL-1β and IL-18**

**Fig. 6:**
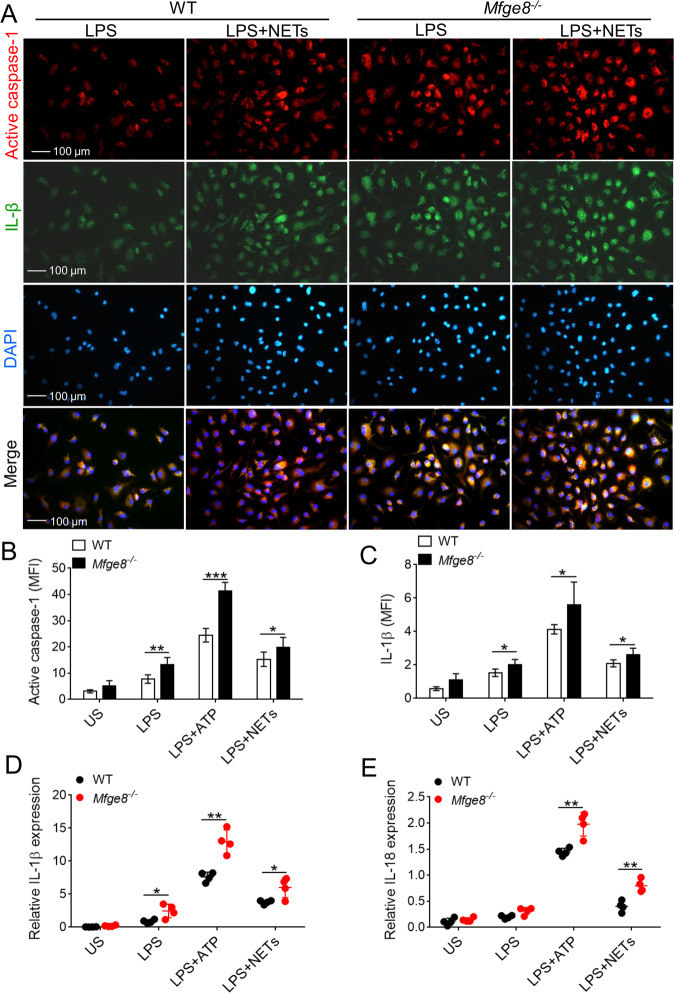
The activation of NLRP3 inflammasome and release of IL-1β and IL-18 were elevated in MFG-E8-deficient macrophages. **a** Representative immunofluorescence images of LPS-primed BMDMs from WT or *Mfge8*^*−/−*^ stained with active caspase-1 and IL-1β after stimulated with 10 μg/mL NETs. The active caspase-1^+^ and IL-1β^+^ BMDMs in five random microscopic fields in *n* = 4 mice per group was performed, original magnification ×400. **b**–**c** Quantified the mean fluorescence density (MFI) of active caspase-1 **b** and IL-1β **c** in LPS-primed BMDMs from WT or *Mfge8*^*−/−*^ mice (*n* = 4) after treatment with NETs or ATP. **d**–**e** Real-time PCR analysis of IL-1β **d** and IL-18 **e** mRNA levels in LPS-primed and ATP or NETs-stimulated WT or *Mfge8*^*−/−*^ BMDMs (from *n* = 4 mice respectively). For all experiments, data are presented as mean ± SEM, *P < 0.05, **P < 0.01, ***P < 0.001.

To determine whether NETs promotes release of IL-1β and IL-18 in MFG-E8-knockout macrophages, similar to other known stimuli of inflammasome, LPS-primed primary WT and *Mfge8*^*−/−*^ macrophages were exposed to control or NETs. After stimulation with NETs, the expression of activated caspase-1 and IL-1β were significantly elevated in *Mfge8*^*−/−*^ macrophages, in addition, ATP-induced caspase-1 activation and IL-1β production were enhanced in LPS-primed *Mfge8*^*−/−*^ macrophages as compared with WT macrophages (Fig. 6a–c). The mRNA levels of IL-1β and IL-18 induced by NETs and ATP in LPS-primed *Mfge8*^*−/−*^ macrophages were remarkably higher than that in WT macrophages (Fig. 6d, e). These data indicate that activated NLRP3 inflammasome targeting IL-18/IL-1β prime neutrophils to NETosis, especially to MFG-E8-deficient neutrophils; the released NETs in turn activate NLRP3 inflammasome and trigger synthesis of IL-18 and IL-1β, especially in MFG-E8-deficient macrophages, resulting in a feed-forward inflammatory loop that could potentially lead to NETs and cytokines IL-18 and IL-1β accumulation in wound tissue and delay healing in MFG-E8-deficient diabetic mice.

### MFG-E8 attenuates NETs-induced IL-18 and IL-1β release

**Fig. 7:**
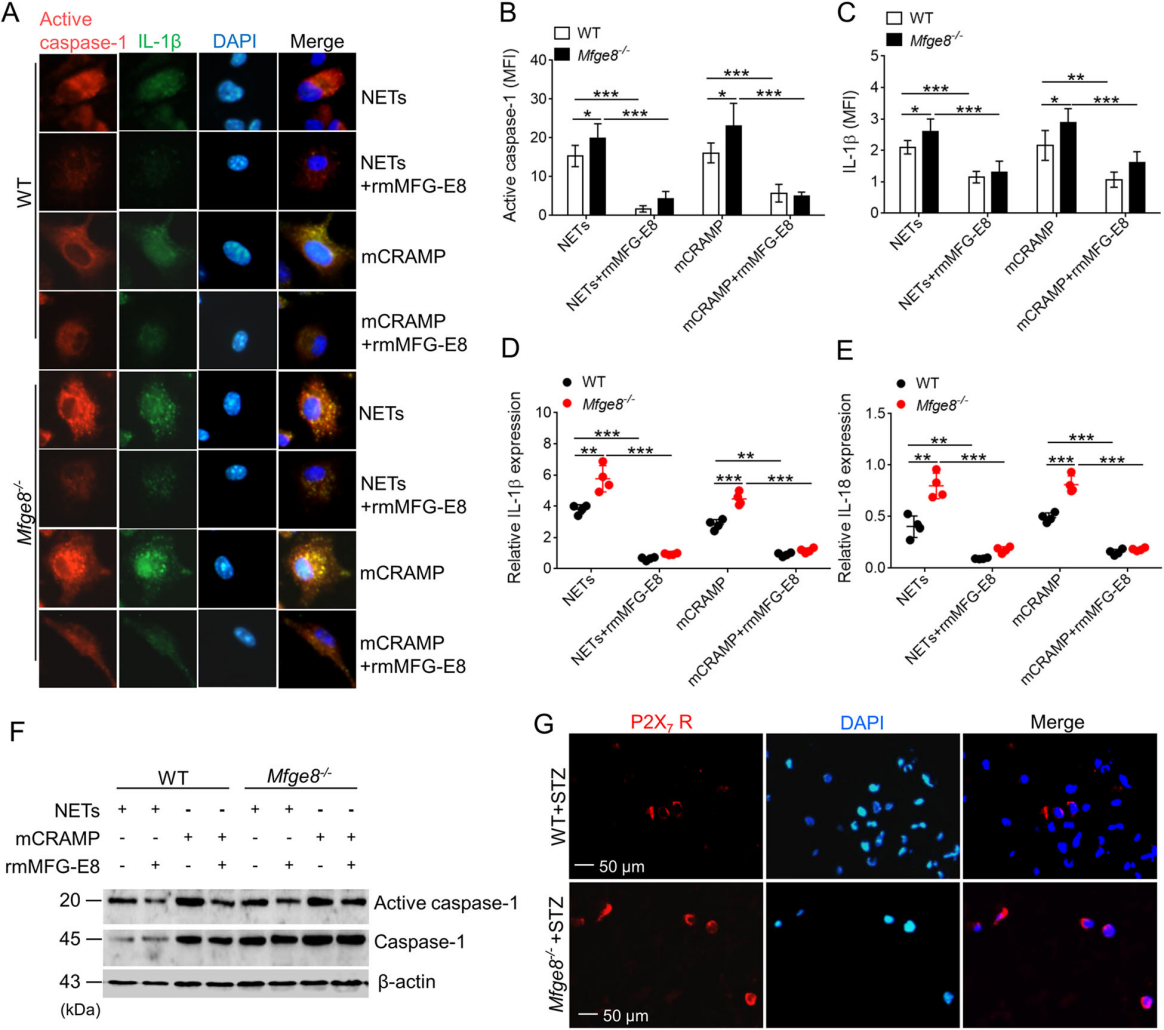
MFG-E8 inhibits the NETs-induced activation of NLRP3 inflammasome. **a** Representative immunofluorescence images of active caspase-1 and IL-1β in LPS-primed BMDMs from WT or *Mfge8*^*−/−*^ mice (*n* = 4) treated with 2 μg/mL mCRAMP (LL-37) or 10 μg/mL NETs, and then administrated with or without rmFMG-E8. The active caspase-1^+^ and IL-1β^+^ BMDMs in five random microscopic fields in *n* = 4 mice per group was performed. **b**–**c** After administration with or without rmMFG-E8, quantified the mean fluorescence density (MFI) of active caspase-1 **b** and IL-1β **c** in LPS-primed BMDMs from WT or *Mfge8*^*−/−*^ mice exposed to NETs or mCRAMP. **d**–**e** Quantification of IL-1β **d** and IL-18 **e** mRNA levels in LPS-primed BMDMs from WT or *Mfge8*^*−/−*^ mice (*n* = 4) after stimulation with NETs or mCRAMP when addition with or without rmMFG-E8. **f** Exposure to NETs or mCRAMP, western blot analysis of the active caspase-1 and caspase-1 expression in BMDMs from WT or *Mfge8*^*−/−*^ mice after treatment with or without rmMFG-E8. **g** The P2X_7_ receptor (P2X_7_ R^+^) macrophages in wound dermis from diabetic WT and *Mfge8*^*−/−*^ mice were determined by immunofluorescence; more than five random microscopic fields in *n* = 6 mice per group was performed. For all experiments, data are presented as mean ± SEM, *P < 0.05, **P < 0.01, ***P < 0.001.

Previous studies showed that NETs are able to activate the NLRP3 inflammasome in a manner that correlates with the concentration of LL-37 in these traps^14^. In this study, we investigated that recombinant mouse CRAMP (the murine equivalent of LL-37 in human) similarly activated caspase-1 and induced IL-1β and IL-18 production (Fig. 7a–c). LPS-primed *Mfge8*^*−/−*^ macrophages displayed significant enhancement in their capacity to release IL-1β and IL-18 following exposure to mCRAMP (Fig. 7d, e), and showed higher expression of activated caspase-1 (Fig. 7b). To assess whether MFG-E8 affect NETs/mCRAMP-induced activation of NLRP3 inflammasome, the rmMFG-E8 was added into NETs or mCRAMP-induced LPS-primed WT or *Mfge8*^*−/−*^ macrophages. Immunofluorescence showed that the expression of activated caspase-1 and IL-1β was significantly reduced in WT and *Mfge8*^*−/−*^ macrophages when treatment with rmMFG-E8 (Fig. 7a–c), consistent with an increase release of IL-1β and IL-18 were in WT and *Mfge8*^*−/−*^ macrophages (Fig. 7d, e). In addition, western blot analysis demonstrated that rmMFG-E8 downregulated the expression of activated caspase-1 in WT and *Mfge8*^*−/−*^ macrophages after stimulation with NETs or mCRAMP (Fig. 7f). These data collectively establish that MFG-E8 has a critical role in suppressing the NETs/mCRAMP-induced NLRP3 inflammasome activation and breaking the “NETs-NLRP3 inflammasome” vicious circle.

LL-37 activation of the NLRP3 inflammasome utilizes P2X_7_ receptor, and MFG-E8 plays a nonredundant role in the control of IL-1β production through integrin β_3_ and limited P2X_7_ receptor^20^. We therefore hypothesized that the inhibition of P2X_7_ receptor was abrogated in wound tissue of MFG-E8-deficient mice after NETs/mCRAMP stimulation. Immunofluorescence assay displayed that the expression of P2X_7_ receptor in wound skin of diabetic *Mfge8*^*−/−*^ mice was enhanced, especially in wound scab site, when compared with that of WT mice after wound for 3 days (Fig. 7g). Thus, MFG-E8 protective effect in NET-primed NLRP3 inflammasome activation requires for intergrin β_3_-limited P2X_7_ receptor pathways.

## Discussion

Our study demonstrated that MFG-E8 promotes wound healing in diabetes by regulating “NLRP3 inflammasome-NETs” axis. The present study provides evidence that inflammatory responses become increasingly amplificative through “NLRP3 inflammasome-NETs” inflammatory loop in wound site of diabetes, and the “NLRP3 inflammasome activation-NETs formation” loop contributes to delayed wound healing in diabetes. Moreover, our data support that MFG-E8 deficiency impairs wound closure and vascularization, and enhances inflammation with NLRP3 inflammasome activation in wound site of diabetes, and diabetes could prime MFG-E8-deficient neutrophils to undergo NETosis. Furthermore, NETs stimulate the activation of NLRP3 inflammasome and release of IL-1β and IL-18, whereas MFG-E8 could attenuate NETs-induced IL-18 and IL-1β release. Hence, the study suggested novel findings regarding the relationship between MFG-E8 and “NLRP3 inflammasome-NETs” inflammatory loop, revealing the modulatory effects of MFG-E8 on “NLRP3 inflammasome-NETs” axis in diabetic wound healing.

MFG-E8 is a glycoprotein that acts as a bridging molecule and interacts with phagocytes to stimulate the uptake of the apoptotic cells, which is ubiquitously expressed in various tissues and cells^15,23^. MFG-E8 has important roles in several biological processes, including apoptotic cell clearance, angiogenesis, and adaptive immunity^23^. In the dermis of normal murine and human skin, accumulations of MFG-E8 colocalized with PDGFRβ^+^, αSMA^+^, and NG2^+^ pericytes around CD31^+^ blood vessels, which promotes cutaneous wound healing by enhancing angiogenesis^24^. Several studies demonstrated that MFG-E8 accelerates wound healing through enhancement of angiogenesis by secretion of pro-angiogenic factors and the differentiation into ECs and/or pericytes, M2 macrophages polarization, improvement of fibroblast migration, and proliferation by production of fibroblast growth factor^16,25,26^. Previous report showed that MFG-E8 promotes resolution of wound inflammation, efferocytosis, improvement of angiogenesis, and accelerates cutaneous wound healing in diabetes^18^. However, the detailed mechanisms of MFG-E8 in regulating wound healing remain largely unknown. Utilizing wound model of STZ-induced diabetic WT and *Mfge8*^*−/−*^ mice, our study is the first to demonstrate that MFG-E8 accelerates angiogenesis and wound closure in diabetes by modulating the “NLRP3 inflammasome-NETs” inflammatory loop.

First, MFG-E8 is an endogenous inhibitor of NLRP3 inflammasome-induced IL-1β/IL-18 production in wound site of diabetes. Previous study elucidated that adoptive transfer of MFG-E8-stimulated macrophages inhibited inflammasome activation and tissue damage^19^. Our data showed higher circulating levels of IL-1β and IL-18 in DFU patients and diabetic wound mice. In diabetic *Mfge8*^*−/−*^ mice, the secretion of IL-1β, IL-18, and TNF-α were significantly increased in circulation and wound site, with an increased recruitment of neutrophils and decreased infiltration of macrophages. Moreover, the activation of NLRP3 inflammation (the expression of active caspase-1), production of IL-1β and IL-18, and pyroptosis were enhanced in wound sites of diabetic MFG-E8-deficient mice.

Second, MFG-E8 inhibits active NLRP3 inflammasome-primed NETs formation in diabetes. In diabetic conditions, neutrophils produce more superoxide and cytokines than normoglycemic conditions^8,27,28^. IL-1β and IL-18 can attract neutrophils, and also prime recruited neutrophils to NETosis, releasing NETs in damaged tissues^13,14,29^. MFG-E8-deficient neutrophils are susceptible to NETosis spontaneously than WT neutrophils. In diabetic *Mfge8*^*−/−*^ mice, increased IL-1β, IL-18, and TNF-α production predipose neutrophils to NETosis. Consistently, IL-18 primed more MFG-E8-deficient neutrophils into NETosis than WT neutrophils. After treatment with rmMFG-E8, the activated NLRP3 inflammasome targeting IL-18-primed NETosis was significantly attenuated in WT or *Mfge8*^*−/−*^ neutrophils. High-dose glucose induces higher NETs when compared to normal glucose or mannitol, indicating a possible priming role of high glucose^3,6^. Given that major insulin-positive islets were broken in STZ-induced MFG-E8 knockout mice, higher level of glucose might be a factor to stimulate neutrophils into NETs. After stimulation with high-dose glucose, *Mfge8*^*−/−*^ neutrophils released higher NETs than those in WT neutrophils in vitro. In addition, our data substantiated the lodged NETs was increased in skin wounds of diabetic *Mfge8*^*−/−*^ mice compared with WT mice, with higher expressions of PAD4, CitrH3, and HMGB-1. NETs and histones directly induce epithelial and endothelial damage^30^, whereas neutrophil elastase can cause degradation of wound matrix and delay healing^31^. Higher NETs located in inflammatory site may explain the slower keratinocyte repopulation in the wound beds of diabetic MFG-E8-deficient mice. These findings suggest that MFG-E8 as NETosis inhibitor is a potential therapeutic strategy for wound-healing acceleration in patients with DFUs.

Third, MFG-E8 dampens the NETs-primed NLRP3 inflammasome activation. Studies reported that NETs are substantially potent in priming IL-1β production and NLRP3 inflammasome activation^14^. NETosis may prime IL-1β production more efficiently than necrosis, as it efficiently exposes highly decondensed and proinflammatory DNA^32^. Thus, the increased NETs formation in wound site of MFG-E8-deficient mice is effective in activating NLRP3 inflammasome mediated by DNA externalization. Except to proinflammatory DNA, macrophages have the potential to internalize LL-37 released from activated neutrophils via P2X_7_ receptor, which activate NLRP3 inflammasome and mediate potassium efflux^14,33,34^. Although ATP has been proposed as the classical ligand for activation of P2X_7_ receptor, LL-37 was concentrated in inflamed areas of high NETosis, which make it a additional plausible ligand for NLRP3 inflammasome pathways. Our data suggested that NETs and mCRAMP (LL-37)-mediated activation of NLRP3 inflammasome was enhanced in MFG-E8-deficient macrophages than that in WT macrophages, releasing higher levels of IL-1β and IL-18. The differences in P2X_7_ receptor expression may contribute to this enhanced activation, and our study showed that the expression of P2X_7_ receptor was elevated in wound skin of diabetic MFG-E8-deficient mice. When treatment with rmMFG-E8, the activation of caspase-1 and production of IL-1β and IL-18 were significantly decreased in WT or *Mfge8*^*−/−*^ macrophages. Thus, MFG-E8 is an important factor in regulating the NETs or LL-37-mediated NLRP3 inflammasome activation.

α_v_β_3_ integrin is one of the main MFG-E8 receptors. MFG-E8 might inhibit the inflammasome pathway through interaction between integrin β_3_ and P2X_7_ receptor. Interestingly, Deroide et al.^20^ reported a low level of colocalization between integrin β_3_ and P2X_7_ receptor in the absence of inflammatory stimuli, while priming macrophages with inflammatory settings increased integrin β_3_ expression and induced tight spatial association between integrin β_3_ and P2X_7_ receptor. Moreover, integrin β_3_-knockout bone marrow-derived macrophages (BMDMs) produce significantly more IL-1β than WT cells in response to ATP stimulation, while MFG-E8 did not alter ATP-induced NLRP3 inflammasome activation and IL-1β production in integrin β_3_-knockout BMDM^20^. However, low expression of integrin β_3_ in wound sites of WT and *Mfge8*^*−/−*^ mice was not detected (data not show). These data indicated that MFG-E8 might regulate NETs-primed inflammasome activation through integrin β_3_, limiting P2X_7_ receptor-dependent NLRP3 inflammasome activation.

In the study, we demonstrated that the levels of MFG-E8 were significantly increased in patients with DFUs compared with healthy controls and diabetic patients, and the expressions of MFG-E8 in skin wound tissues of diabetic mice were enhanced when compared with normal mice. However, under diabetic conditions, hyperglycemia or exposure to glycated end products may cause MFG-E8 glycation that leads to structural and functional changes of MFG-E8. Das et al.^18^ showed that MFG-E8 gets glycation in diabetic ulcers, and glycated MFG-E8 displays weaker binding to phosphatidylserine (PS) as compared with the binding of nonglycated MFG-E8 to PS. Therefore, glycation has a major impact on MFG-E8 function despite high levels of MFG-E8 in DFU patients and diabetic wound mice, suggesting that not only the amount but also the functionality of MFG-E8 is a key point to be addressed. Thus, the exogenous MFG-E8 harbors therapeutic potential in resolving the dysfunction of endogenous glycated MFG-E8.

In conclusion, our work identified MFG-E8-mediated protective mechanism of wound injury in diabetes, including: (i) suppression of activated NLRP3 inflammasome targets IL-1β/IL-18 synthesis; (ii) attenuation of NETs release from neutrophils induced by inflammatory cytokine IL-1β/IL-18; (iii) might control NET-primed NLRP3 inflammasome activation through integrin β_3_ and limiting P2X_7_ receptor; (iv) enhancement of angiogenesis in wound bed. Taken together, as master of key hub in regulating the “NLRP3 inflammasome-NETs” loop, MFG-E8 induced resolution of wound inflammation, decreased NETs accumulation, improved angiogenesis, and accelerated wound closure, supporting that exogenous MFG-E8 administration has possible therapeutic potential for diabetic wound.

## Methods

### Patients

Three groups of individuals were enrolled: 30 healthy control subjects, 33 patients with diabetes, and 25 patients with DFUs (age range 21–65 years old). Subjects with diabetes were recruited only if they were not on steroid or other immunosuppressive medications, not presenting any signs of active infection (fever, high leukocytes count, and diagnosis of infection), without diagnosis of cancer in the past 5 years and without overt heart failure. The sera from three groups were used to detect the levels of neutophil elastase, proteinase 3, IL-1β, IL-18, and MFG-E8. Neutrophils were isolated from peripheral blood of 10 healthy controls, 12 patients with diabetes, and 12 patients with DFUs using Percoll gradients and primed to NETosis spontaneously. Clinical and serological information was obtained from the patients’ medical records. This study was conducted according to Declaration of Helsinki, and ethical and experiment approval was obtained from the Research Ethics Committee of Wuhan No.1 Hospital (Wuhan, China). Informed consent was also obtained from all subjects.

### Human neutrophil isolation and NETosis assay

Neutrophils were isolated from peripheral blood of 10 healthy controls, 12 diabetic patients, and 12 DFU patients using Neutrophils isolation kit (Solarbio, Beijing, China). Purity of cells was >95% as determined by Wright–Giemsa staining. Neutrophils were resuspended at 2 × 10^5^ cells per well in RPMI 1640 medium (Hyclone, Thermo Fisher, MA, USA) and plated onto poly-l-lysine-coated coverslips (Sigma-Aldrich, MO, USA) in 24-well culture plates (Costa, Cambridge, USA), which were incubated in serum-free medium for 2 h to prime NETosis spontaneously. Cells were then instantly fixed in 4% paraformaldehyde (PFA) for NET quantification as previously described^21^. NETs were stained with anti-citrullinated histone H3 (CitrH3, citrulline R2 + R8 + R17, Abcam ab5103, Cambridge, USA) and anti-MPO (Abcam ab90810) antibodies, followed by incubation with secondary Alexa Flour 488 (Abcam ab150077) and Alexa Flour 647-conjugated antibodies (Abcam ab150115). After DNA was stained with 4′,6-diamidino-2-phenylindole (DAPI, Sigma-Aldrich), images were collected with Olympus BX51 microscope (Olympus, Tokyo, Japan) and Qimaging camera (RoHs, British Columbia, Canada). Percentage of NETs was determined from five non-overlapping fields per well for every sample, and then the images were merged with Image J software (NIH, MD, USA).

### **Animal model of diabetes**

*Mfge8*^*−/−*^ mice were constructed in a C57BL/6 background by deleting an exon III to exon IIV genomic fragment generated by Nanjing Biomedical Research Institute of Nanjing University (NBRI, Nanjing, China). WT (C57BL/6) and *Mfge8*^*−/−*^ mice were maintained under specific pathogen-free conditions at the animal housing facility at Wuhan No.1 Hospital. Diabetes was induced in 8-week-old male mice by fasting for 5 hours and then i.p. injection of 100 mg/kg STZ (Sigma-Aldrich) in 1 mm citrate buffer (pH 4.5) for 5 days. Vehicle (citrate buffer)-injected animals served as controls. The weight was measured at 0, 3, 7, 14, 28 day after injection. After 4 weeks for treatment with STZ, fed glucose levels in peripheral blood were measured with a portable glucometer (Roche, Indianapolis, USA). Animals with blood glucose levels >300 mg/dL were considered diabetic and used for subsequent experiments^3^. The studies involving animal model were conducted consulting the ARRIVE guidelines, and were approved by the ethics committee of Wuhan No. 1 Hospital.

### Dorsal excisional wound model

A full-thickness excisional wounds were made on the backs of WT (*n* = 24), diabetic WT (*n* = 24), *Mfge8*^*−/−*^ mice (*n* = 24), and diabetic *Mfge8*^*−/−*^ mice (*n* = 24), who were randomly assigned to four groups (at day 0, 3, 7 14 post-wounding), as previously described^18^. In brief, the dorsal side of the mice was naired and cleaned using betadine under anesthesia. A 6-mm-diameter full- thickness (skin and panniculus carnosus) excisional wounds were made on the dorsal skin with a 6-mm disposable biopsy punch. The wound area was calculated from the photographs at day 0, 3, 7 14 postwounding using Image J analysis software (NIH) and expressed as a percentage of the original wound area. For detection of wound leukocytes and cytokines, circular (8 mm) sterile polyvinyl alcohol sponges were implanted s. c. on the backs of diabetic WT or *Mfge8*^*−/−*^ mice^35^. Sponge-infiltrated wound leukocytes and cytokines were isolated at day 0, 3, 7, and 14 post-wounding, as previously described^35^. Animal studies were blinded during the group allocation, experiments and when assessing the outcomes.

### **Histopathology and TUNEL assay**

Excised wound tissues were fixed immediately with 4% PFA for histological analysis. Sections (5 μm thick) were prepared from paraffin-embedded tissues and subjected to hematoxylin and eosin staining. The presence of death cells in the wound skin sections were assessed after wound for 3 days using terminal deoxynucleotide transferase dUTP nick end-labeling (TUNEL) staining kit (Roche Diagnostics, Indianapolis, USA) according to the manufacturer’s instruction. The sections were examined with Olympus BX51 microscope and Qimaging camera, and the number of apoptotic cells was determined by counting TUNEL and DAPI double positive nuclei in the field (×400).

### Flow cytometry

Sponge-infiltrated wound leukocytes was filtered through a 70-μm nylon filter (BD Biosciences, CA, USA), centrifuged at 1200 × *g* for 5 minutes, and washed with phosphate-buffered saline (PBS) containing 0.5% bovine serum albumin (BSA). The cells were labeled with fluorescein isothiocyanate-labeled anti-mouse CD11b antibody (BD Biosciences 553310, USA), PE-labeled anti-mouse Ly6G antibody (BD Biosciences 551461), and phycoerythrin-cyanine 5-labeled anti-mouse F4/80 antibody (eBiosciences 15–4801, Thermo Fisher, MA, USA). After washing, the cells were analyzed by flow cytometry (FACSCalibur; BD Biosciences). The data were analyzed by FlowJo software (Tree Star, CA, USA) with 10,000 events per sample.

### ELISA

The serum IL-18 (Abcam), IL-1β (R&D Systems, MN, USA), neutrophil elastase (R&D Systems), proteinase 3 (R&D Systems), and MFG-E8 (Abcam) levels in 30 healthy controls, 33 diabetic patients and 25 DFUs were detected with human ELISA kits. After wound, the serum levels of TNF-α (eBioscience), IL-1β (eBioscience), IL-18 (Abcam), and MFG-E8 (R&D Systems) in diabetic WT and *Mfge8*^*−/−*^ mice at day 0, 3, 7, 14 postwounding were detected with ELISA kits. Then, the levels of IL-1β (eBioscience), TNF-α (eBioscience), IL-18 (Abcam), and IL-10 (R&D Systems) in wound fluid of WT and *Mfge8*^*−/−*^ mice after wound for 3 days were measured using commercially available ELISA kits.

### Mouse neutrophil isolation and NETosis assay

WT and *Mfge8*^*−/−*^ neutrophils were isolated from bone marrow cells using Percoll (Sigma-Aldrich) gradients (55%, 62%, and 81% Percoll in PBS). Cells at the 62/81% interface were extracted and identified the purity of cells was >95% as determined by Wright–Giemsa staining. Identification of NETs as previous described^21^. Neutrophils were resuspended in Hanks' Balanced Salt Solution (with calcium, magnesium, and 5.5 mm glucose) for experiments involving high glucose; otherwise they were resuspended in RPMI 1640 medium (Hyclone). In all, 2 × 10^5^ neutrophils were plated onto poly-l-lysine-coated coverslips (Sigma-Aldrich), and stimulated with 2% serum from WT or *Mfge8*^*−/−*^ mice at 3 day postwounding, 1 μg/ml LPS (Sigma-Aldrich), 100 nm PMA (Abcam) or 25 ng/ml IL-18 (BioVison 7326–100, CA, USA) in serum-free RPMI 1640 medium (Hyclone) for 2 h. For high glucose experiments, neutrophils were isolated from WT and *Mfge8*^*−/−*^ mice and incubated for 2 h in media with normal (5.5 mm) or high (22 mm) glucose concentrations. For determination the effect of recombinant mouse MFG-E8, 500 ng/mL rmMFG-E8 (R&D Systems P21956) and 25 ng/mL IL-18 were simultaneously added into serum-free RPMI 1640 medium with plated WT and *Mfge8*^*−/−*^ neutrophils and incubated for 2 h. Then the NETs were determined with anti-CitrH3 (Abcam), anti-MPO (Abcam) antibodies and DAPI (Sigma-Aldrich). The images were acquired with Olympus BX51 microscope and Qimaging camera typically at original ×400 magnification, and the percentage of NETs were determined from 5 to 6 non-overlapping fields per well and the average was taken from triplicates for each condition in every experiment.

### Mouse BMDMs isolation and treatment

WT or *Mfge8*^*−/−*^ mice BMDMs were obtained by isolating bone marrow from tibias and femurs by flushing with RPMI 1640 medium, followed by growth in RPMI 1640 medium (Hyclone) supplemented with 20 ng/mL M-SCF (PeproTch #315-02, New Jersey, NJ), 10% heat-inactivated FBS (Gibco, Thermo Fisher, MA, USA), 2 mm penicillin and streptomycin (Invitrogen, Thermo Fisher, MA, USA). Cells were cultures for a week with media changes every 3 days. For quantification of inflammasome activation, macrophages were primed with 100 ng/mL LPS (Sigma-Aldrich) for 4 h prior to stimulation. Media were then removed and replaced with phenol red-free, serum-free RPMI 1640 prior to treatment with 5 mm ATP (Sigma-Aldrich) or 2 μg/mL mouse cathelicidin (mCRAMP ab104417, Abcam) for 2 h. For experiments using NETs, neutrophils were primed to NETosis after sitmulation with 100 nm PMA (Abcam) and collected culture supernatants. The concentration of NETs protein were determined with Pierce ®BCA Protein Assay kit (Thermo Pierce, Thermo Fisher, MA, USA). 10 μg/mL NETs were added into WT or *Mfge8*^*−/−*^ BMDMs for 2 h. When treatment with rmMFG-E8, WT or *Mfge8*^*−/−*^ BMDMs were primed prior to 100 ng/mL for 4 h and then stimulated with 2 μg/mL mCRAMP (Abcam) and treated with 500 ng/mL rmMFG-E8 simultaneously for 2 h. Following treatment, cells were fixed with 4% PFA, blocked with 5% BSA, and incubated with anti-IL-1β (R&D Systems AF-401-NA), anti-caspase-1 p20 antibodies (active caspase-1, Santa Cruz #SC-1218, TX, USA). Then secondary Alexa Flour 488 and Alexa Flour 647-conjugated antibodies (Abcam) were stained. After labeled with DAPI (Sigma-Aldrich), images were obtained with Olympus BX51 microscope and Qimaging camera typically at original ×400 magnification. Moreover, cells were collected for quantification of IL-1β and IL-18 by real-time PCR, and measure of active caspase-1, caspase-1 by western blot.

### Western blot

Proteins from the wound skin tissues of WT mice treated with vehicle or STZ were separated by sodium dodecyl sulfate polyacrylamide gel electrophoresis (SDS-PAGE) and detected with goat anti-mouse MFG-E8 antibody (R&D Systems AF2805). Levels of PAD4, CitrH3, HMGB-1, MFG-E8, cleaved caspase-1, caspase-1 in skin tissues of diabetic WT or *Mfge8*^*−/−*^ mice after wound for 3 days were quantified by western blot. The expression of caspase-1, active caspase-1 in stimulated WT or *Mfge8*^*−/−*^ BMDMs were determined with western blot. After separated by SDS-PAGE, proteins were incubated with anti-MFG-E8 (R&D systems AF2805), anti-PAD4 (Abcam ab214810), anti-citrullinated histone H3 (Abcam ab5103), anti-HMGB-1 (Abcam ab77302), cleaved caspase-1 (Cell Signaling Technology #67314), or caspase-1 (Abcam ab108362) antibodies. Signal was visualized using corresponding horseradish peroxidase-conjugated secondary antibodies (Southern Biotech; 1:4000) and ECL Plus enhanced chemiluminescence kit (Thermo Pierce). Equal loading was confirmed by probing for β-actin or GAPDH (Sigma-Aldrich G9545; 1:5000). Blots were quantified using Image Lab software (Bio-Rad Laboratories, CA, USA).

### Immunofluorescence

For immunofluorescence assay, sections of paraffin-embedded wound skin tissues from WT or *Mfge8*^*−/−*^ mice were deparaffinized and subjected to antigen retrieval. After permeabilized with 0.03% Triton X-100, and blocked in 5% BSA, anti-IL-1β (Abcam), anti-caspase-1 p20 (active caspase-1, Santa Cruz #SC-1218, Cell Signaling Technology, MA, USA), anti-CD31 (Abcam ab28364), and anti-α-smooth muscle actin (α-SMA) antibodies (R&D Systems MAB1420) were incubated overnight at 4°C. Secondary Alexa Flour 488 or Alexa Flour 647-conjugated antibodies (Abcam) were added for 2 h and then stained with DAPI (Sigma-Aldrich). For NETs assay, skin tissues sections were stained using anti-CitrH3 and anti-MPO antibodies (Abcam), followed by Alexa Fluor-conjugated secondary antibodies, and DAPI (Sigma-Aldrich). For P2X_7_ receptor detection, skin tissues were incubated with anti-P2X_7_ receptor antibody (Abcam ab195356), and following with Alexa Flour 647-conjugated antibodies. Sections (×200 or ×400) were imaged with Olympus BX51 microscope and Qimaging camera, and more than five fields per section were obtained.

### Real-time PCR

To analyze the mRNA levels of IL-1β and IL-18 in WT or *Mfge8*^*−/−*^ BMDMs by real-time PCR, the stimulated cells were collected. Total RNA was isolated by Trizol (Invitrogen, Thermo Fisher, MA, USA) and was subjected to reverse transcription using ReverTra Ace®qPCR RT kit (TOYOBO, Osaka, Japan) according to the manufacturer’s instructions. Quantitative real-time PCR was performed with SYBR® Green Real-time PCR Master Mix (TOYOBO) using AB 7500 instrumentation (Applied Biosystems, CA) according to the manufacturer’s instructions. Primer sequences for the IL-1β: 5′-TCATTGTGGCTGT GGAGAAG-3′ and 5′-AGGCCACAGGTATTTTGTCG-3′; IL-18: 5′-GCCATGTCAGA AGACTCTTGCGTC-3′ and 5′-GTACAGTGAAGTCGGCCAAAGTTGTC-3′; β-actin: 5′-GTGGGCCGCTCTAGGCACCAA-3′ and 5′-CTCT TTGATGTCACGCACGATTTC-3′. The quantification of results was performed by the comparative Ct (2^–ΔΔCt^) method. The Ct value for each sample was normalized to the value for the β-actin gene.

### Statistical analysis

All results are reported as mean±SEM, and numbers of experiments (*n*) are indicated. Statistical tests are justified as appropriate for every figure, and the data meet the assumptions of the tests, including normal distribution. The estimated variance is similar between the groups that are being statistically compared. Two-tailed Student’s *t* test was used to determine the significance of the differences using SPSS 16.0 (SPSS, IBM, NY, USA) and GraphPad Prism software 5.0 (GraphPad, CA, USA). A *P* value of < 0.05 was considered statistically significant.

## Supplementary information

Supplemental Figure S1

Supplemental Figure S2

Supplemental Figure S3

Supplementary Figure Legends

Supplemental Table S1

Supplemental Experimental Procedures
