# Supplementary figures and images for: MFG-E8 accelerates wound healing in diabetes by regulating “NLRP3 inflammasome-neutrophil extracellular traps” axis
Source: Cell Death Discov. 2020 Sep 10;6:84. doi: 10.1038/s41420-020-00318-7 (PMC7484765; doi:10.1038/s41420-020-00318-7)

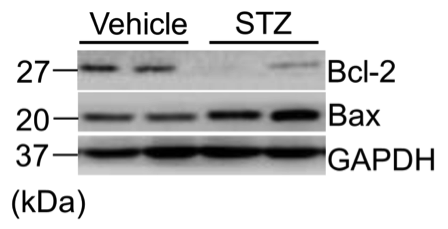

Supplement: Supplementary file 1 — Supplemental Figure S1 [file 41420_2020_318_MOESM1_ESM.png]

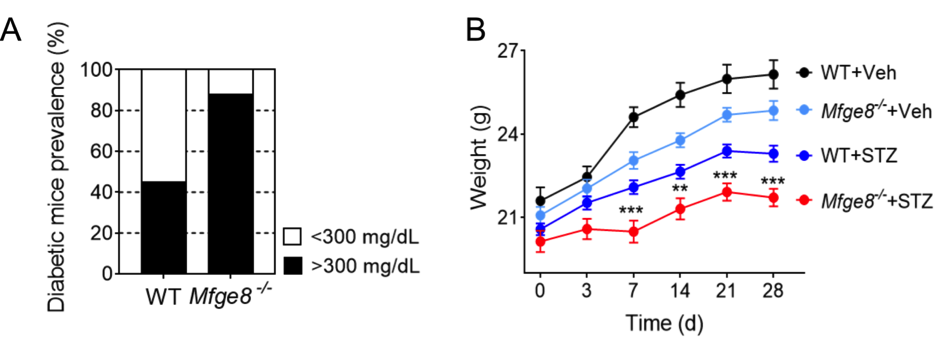

Supplement: Supplementary file 2 — Supplemental Figure S2 [file 41420_2020_318_MOESM2_ESM.png]

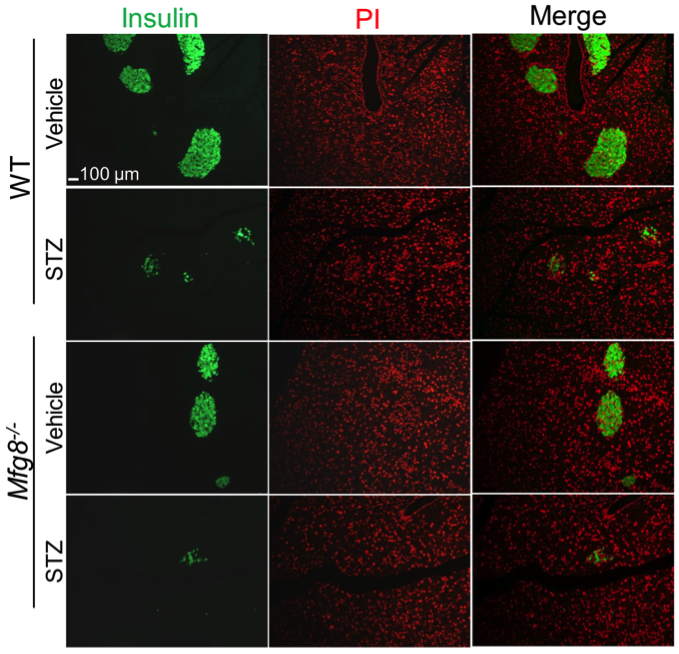

Supplement: Supplementary file 3 — Supplemental Figure S3 [file 41420_2020_318_MOESM3_ESM.png]
