# Supplementary material for: MFG-E8 accelerates wound healing in diabetes by regulating “NLRP3 inflammasome-neutrophil extracellular traps” axis
Source: Cell Death Discov. 2020 Sep 10;6:84. doi: 10.1038/s41420-020-00318-7 (PMC7484765; doi:10.1038/s41420-020-00318-7)
Supplement: Supplementary file 4 — Supplementary Figure Legends [file 41420_2020_318_MOESM4_ESM.docx]

**Figure S1** **The expression of apoptosis-related protein in wound diabetic mice.** The apoptosis-related Bax and anti-apoptosis-related Bcl-2 expression in skin tissue of WT and STZ-induced diabetic WT mice (n=6) at day 3 postwounding, GAPDH as a loading control.

**Figure S2 The susceptibility of STZ-induced diabetes in MFG-E8-deficicent mice.** (**A**) The ratio of diabetic WT (n=24) and *Mfge8^-/-^* mice (n=28) were calculated, and the serum fed glucose concentration >300 mg/dL as diabetic mice. (**B**) The weight of WT (n=24) or *Mfge8^-/-^* mice (n=28) injected vehicle or STZ were weighed at 0, 3, 7, 14, 21, and 28 days. data are presented as mean ±SEM, ^*^*^*^P*<0.01, ^**^*^*^P*<0.001 vs WT mice induced by STZ.

**Figure S3 The insulin-positive islets were broken in STZ-induced diabetic MFG-E8-deficient mice.** The expression of insulin in islets of WT (n=6) or *Mfge8^-/-^* mice (n=6) after treatment with vehicle or STZ was detected with immuno-fluorescence.
