# Supplementary material for: MFG-E8 accelerates wound healing in diabetes by regulating “NLRP3 inflammasome-neutrophil extracellular traps” axis
Source: Cell Death Discov. 2020 Sep 10;6:84. doi: 10.1038/s41420-020-00318-7 (PMC7484765; doi:10.1038/s41420-020-00318-7)
Supplement: Supplementary file 5 — Supplemental Table S1 [file 41420_2020_318_MOESM5_ESM.docx]

**Table S1. Clinical characteristics of patients in the validation cohort.**

| **Characteristics** | **Healthy controls** | **Diabetes** | **DFUs** |
| --- | --- | --- | --- |
| Number | 30 | 33 | 25 |
| Age (years) | 57.0 ± 1.90 | 57.5 ± 2.70 | 58.0 ± 2.00 |
| Male sex (%) | 53.30 | 66.70 | 64.00 |
| FBG (mmol/L) | 4.68 ± 0.08 | 13.56 ± 0.87^*^ | 12.94 ± 0.96^*^ |
| PG2h (mmol/L) | - | 19.87 ± 0.97 | 16.18 ± 1.11^#^ |
| HbA1c (%, mmol/mol) | 5.3 ± 0.04 | 10.5 ± 0.70^*^ | 8.5 ± 0.40^*^ |
| Insulin (pmol/L) | - | 51.88 ± 9.47 | 82.55 ± 25.46 |
| C-peptide (nmolL) | - | 0.87 ± 0.30 | 0.59 ± 0.08 |
| WBC (×10^9^/L) | 5.52 ± 0.23 | 6.12 ± 0.28^*^ | 8.11 ± 0.75^*^ |
| Neutrophil % | 54.9 ± 1.1 | 54.9 ± 1.60 | 69.5 ± 2.40^*#^ |
| Monocyte % | 6.2 ± 0.30 | 8.3 ± 0.40^*^ | 9.1 ± 0.60^*^ |
| Neutrophil (×10^9^/L) | 3.05 ± 1.16 | 3.42 ± 0.22 | 5.82 ± 0.68^*#^ |
| Monocyte (×10^9^/L) | 0.34 ± 0.03 | 0.49 ± 0.02^*^ | 0.71 ± 0.06^*#^ |

*^*^P*<0.05 vs. healthy controls. *^#^P*<0.05 vs. patients with diabetes. FBG, fasting blood- glucose; PG2h, 2-hour postprandial blood glucose; WBC, white blood cell.
