# Supplementary material for: MFG-E8 accelerates wound healing in diabetes by regulating “NLRP3 inflammasome-neutrophil extracellular traps” axis
Source: Cell Death Discov. 2020 Sep 10;6:84. doi: 10.1038/s41420-020-00318-7 (PMC7484765; doi:10.1038/s41420-020-00318-7)
Supplement: Supplementary file 6 — Supplemental Experimental Procedures [file 41420_2020_318_MOESM6_ESM.docx]

**Western Blot**

Proteins from the wound skin tissues of WT mice treated with vehicle or STZ were separated by SDS-PAGE and detected with rabbit anti-mouse Bax (Cell Signaling Technology #14796), and Bcl-2 antibody (Cell Signaling Technology #14796). Signal was visualized using corresponding HRP-conjugated secondary antibodies (Southern Biotech; 1:4000) and ECL Plus enhanced chemiluminescence kit (Thermo Pierce). Equal loading was confirmed by probing for GAPDH (Sigma-Aldrich G9545; 1:5000). Blots were quantified using Image Lab software (Bio-Rad Laboratories, CA).

**Immunofluorescence**

After injection of STZ for 28 days, pancreatic islets were stained for insulin using a rabbit polyclonal anti-insulin antibody (1: 400, Cell Signalling Technology #8138), followed by Alexa Flour 488-conjugated secondary antibody (1:1000, Abcam ab150077) and DAPI (Sigma-Aldrich). Images were acquired on Olympus BX51 microscope and Qimaging camera.
